# Study on pathogen monitoring and critical patient treatment strategies for acute community-acquired lower respiratory tract infections

**DOI:** 10.12669/pjms.41.9.12121

**Published:** 2025-09

**Authors:** Ke Gong, Siqi Yang

**Affiliations:** 1 Ke Gong Department of Thyroid and Breast Surgery, Changde Hospital, Xiangya School of Medicine, Central South University, The first people’s hospital of Changde City, Changde 415000, Hunan, China; 2 Siqi Yang Department of Ministry of Public Health, Changde Hospital, Xiangya School of Medicine, Central South University, The first people’s hospital of Changde City, Changde 415000, Hunan, China

**Keywords:** Acute community-acquired lower respiratory tract infection, Critically ill patient, Pathogen distribution, Treatment strategy

## Abstract

**Objective:**

To investigate the characteristics of pathogen distribution in patients with acute community-acquired lower respiratory tract infections(ACALRTIs) and treatment strategies for critically ill patients.

**Methodology:**

This was a retrospective study. Clinical data were collected from 218 patients diagnosed with ACALRTIs who received treatment at Changde Hospital, Xiangya School of Medicine, Central South University (The first people’s hospital of Changde city) between January, 2022 to June, 2024. Based on the presence or absence of complications during treatment, patients were divided into an observation group without complications and a control group with complications. The clinical treatment outcomes, clinical symptoms, blood gas analysis indicators were compared between the two groups.

**Results:**

The sputum smear positivity rate in the observation group was 75.68%, significantly higher than the 54.65% observed in the control group(*P<* 0.05). However, there was no significant difference in sputum culture positivity between the two groups (*P>* 0.05). The overall response rate in the observation group was 94.00%, significantly higher than the 84.75% in the control group (*P<* 0.05). After treatment, the observation group had a shorter time to fever resolution, cough resolution, and disappearance of pulmonary wheezing compared to the control group (*P<* 0.05, respectively). The observation group also showed higher SaO_2_, and PaO_2_, levels, while PaO_2_, PCT, WBC, CRP, and ESR levels were lower than those in the control group (*P<* 0.05, respectively).

**Conclusion:**

ACALRTIs are primarily caused by bacterial, viral, and atypical pathogens. The use of bronchoalveolar lavage under fiberoptic bronchoscopy may improve blood gas parameters and inflammatory marker levels, alleviate clinical symptoms.

## INTRODUCTION

Community-acquired lower respiratory tract infections (CALRTIs) are among the leading infectious diseases causing death. CALRTIs are common and frequently occurring in clinical practice, with acute onset being the most prevalent form. These infections result in approximately four million deaths globally each year. The main pathogens responsible for CALRTIs are viruses, bacteria, and atypical pathogens.^1^,^2^ Due to variations in healthcare conditions, regional environments, and economic factors, the causative pathogens of CALRTIs exhibit distinct regional characteristics. In clinical treatment, accurately identifying the pathogens not only holds significant epidemiological and public health value, but also allows for targeted, precise treatment, which can markedly improve patient prognosis.^3^,^4^

However, the pathogen spectrum of CALRTIs has gradually changed due to several factors, including the widespread use of antibiotics, the application of vaccines, an aging population, increased complications, climate change, and the prevalence of respiratory infectious diseases. Currently, less than half of patients with acute CALRTIs (ACALRTIs) receive a clear pathogen diagnosis. Particularly in recent decades, multiple emerging and re-emerging respiratory infectious diseases have led to outbreaks and epidemics, such as severe acute respiratory syndrome coronavirus (SARS-CoV), avian influenza virus HSN1 subtype (H5N1), Middle East respiratory syndrome coronavirus (MERS-CoV), influenza A virus H1N1 subtype (H1N1), and severe acute respiratory syndrome coronavirus 2 (SARS-CoV-2), which has caused a global pandemic in the last three years, placing a tremendous burden on society.

Therefore, it is clinically critical to analyze the pathogen distribution patterns in patients with CALRTIs and provide critically ill patients with CALRTIs with timely, effective treatments that improve clinical outcomes and reduce mortality.^5^,^6^ This requires the establishment of appropriate treatment strategies to quickly and professionally manage critically ill patients with CALRTIs and improve clinical treatment success rates. This study investigated the pathogen distribution and treatment strategies for patients with CALRTIs treated at Changde Hospital, Xiangya School of Medicine, Central South University (The first people’s hospital of Changde city), providing scientific evidence for the treatment of critically ill patients with CALRTIs.

## METHODS

This retrospective study collected clinical data from 218 patients with ACALRTIs who received treatment at Changde Hospital, Xiangya School of Medicine, Central South University (The first people’s hospital of Changde city) between January 2022 to June 2024. Based on the presence or absence of complications during treatment. The 218 patients were divided into an observation group (100 patients without complications) and a control group (118 patients with complications).

### Ethical approval:

The study was approved by the Institutional Ethics Committee of Changde Hospital, Xiangya School of Medicine, Central South University (The first people’s hospital of Changde city) (No.: 2023-015-03; date: March 28, 2024), and written informed consent was obtained from all participants.

### Inclusion criteria:

- Meeting the diagnostic criteria for ACALRTIs.
- Complete clinical data.

### Exclusion criteria:

- Speech impairments or concomitant mental illnesses.
- Congenital heart disease.
- Other infectious diseases, such as cardiopulmonary resuscitation, trauma, postoperation, burn, shock, sunstroke, neuroendocrine neoplasm, extracorporeal circulation, liver cirrhosis, pancreatitis, mesenteric necrosis and catheter infections.
- Incomplete clinical data.

Nasopharyngeal swabs, sputum, blood, and urine samples were collected from patients within 72 hours of admission. Additional clinical specimens included pleural fluid, tracheal aspirates, and alveolar lavage fluid. The sputum was stored at 4°C and immediately transported to the laboratory for treatment within two hours. The sputum was diluted and centrifuged to obtain the supernatant and stored at -70°C until cytokine levels were evaluated. The Pathogeno Lung-Q respiratory multiple test kit was used to test nasopharyngeal swabs, sputum, tracheal aspirates, and alveolar lavage fluid for a range of pathogens, including influenza A, influenza B, parainfluenza, adenovirus, coronavirus, *Streptococcus pneumoniae*, *Haemophilus influenzae*, and Mycoplasma pneumoniae. All lower respiratory tract and pleural fluid specimens were tested using smear, culture, and polymerase chain reaction in combination.

In addition to actively treating the underlying disease, the control group was administered anti-infection therapy, bronchodilators and antispasmodics, expectorants and cough suppressants, fluid resuscitation, and maintenance of electrolyte balance. In the observation group, bronchoalveolar lavage under fiberoptic bronchoscopy (BAL-FOB) was performed in addition to the treatment used in the control group. Specifically, the patient inhaled pure oxygen for five minutes while monitoring blood oxygen saturation (SaO_2_) and electrocardiogram. If the patient became agitated, 10 mg of diazepam was administered intramuscularly, or 3–5 mL of propofol was injected intravenously. The airway was anesthetized with 5-mg of 2% lidocaine, and a bronchoscope was inserted through the nose into the bronchus. The bronchial secretions were aspirated, and the bronchi and alveoli were washed with 10–20 ml of 37°C saline via the lavage channel, performing small-volume, multiple lavages until the lavage fluid became clear. About 20 ml of antibiotic dilution was then infused, and after the lavage, expiratory positive pressure ventilation was applied. Lavage was repeated every 2–3 days, with antibacterial therapy selected based on the results of lavage fluid cultures, and a total of 3–5 lavages were performed.

### Efficacy and outcome measures:

The treatment efficacy was determined based on patients’ condition after treatment, comparing the clinical outcomes between the two groups. Clinical efficacy was classified as follows:

### Significant Response (SR):

After treatment, temperature, white blood cell count (WBC), and other parameters returned to normal, with significant alleviation in symptoms related to lung infection, and chest X-rays showed that the infection foci had essentially disappeared.

### Partial Response (PR):

After treatment, symptoms related to lung infection were partially relieved, and chest X-rays showed a significant reduction in the infection foci.

### No Response (NR):

After treatment, symptoms did not improve, and chest X-rays showed no reduction in the infection foci. Overall response rate (ORR) = (SR cases + PR cases) Total cases ×100%.

### Outcome measures are as follows:

- ***Clinical Symptom Improvement Time:*** Time to fever resolution, time to cough resolution, and time to disappearance of pulmonary wheezing.
- ***Blood Gas Parameters:*** SaO_2_, partial pressure of oxygen (PaO_2_), and partial pressure of carbon dioxide (PaCO_2_,).
- ***Serum Inflammatory Markers:*** Procalcitonin (PCT), WBC, C-reactive protein (CRP), and erythrocyte sedimentation rate(ESR).

### Statistical analysis:

Data were statistically analyzed using SPSS22.0. The confidence interval is 95%, measurement data were expressed as mean ± standard deviation (*X̅*±*S*), and intergroup comparisons were performed using the t-test. Categorical data were expressed as frequencies or percentages (*n*[%]), and comparisons between groups were made using the chi-square (*χ*²) test. A *P*-value of <0.05 was considered statistically significant.

## RESULTS

There were no statistically significant differences between the two groups in sex, age, time interval between symptom onset and admission, presence of fever, and presence of respiratory distress (all *P >* 0.05), suggesting a high degree of comparability (Table-I).

**Table-I:** Comparison of general clinical data between the two groups.

Group	n	Sex (n)		Age (years)	Time interval between symptom onset and admission (*X̄*±*S*)	Fever (Presence/Absence, n)	Respiratory distress (Presence/Absence, n)
		Male	Female				
Observation group	100	54	46	59.28±17.49	4.38±2.09	65/35	61/39
Control group	118	79	39	63.38±17.51	4.49±2.23	89/29	60/58
t/χ² value		0.054		0.244	0.378	2.836	2.259
P-value		0.816		0.808	0.706	0.092	0.133

After treatment, there were two deaths in the control group, while no deaths occurred in the observation group. The difference was not statistically significant (*χ*² = 1.711, *P =* 0.191). The ORR in the observation group was 94.00%, significantly higher than the 84.75% in the control group (*P <* 0.05) (Table-II).

**Table-II:** Comparison of clinical efficacy between the two groups (n[%]).

Group	n	SR	PR	NR	ORR (%)
Observation group	100	56(50.00)	38(42.50)	6(7.50)	94(94.00)
Control group	118	53(44.92)	47(39.83)	18(15.25)	100(84.75)
*χ²*-value					4.732
*P*-value					0.030

In the observation group, 74 patients underwent sputum smear testing, and 72 patients underwent sputum culture. In the control group, 86 patients underwent sputum smear testing, and 81 patients underwent sputum culture. The sputum smear positivity rate in the observation group was 75.68%, significantly higher than the 54.65% in the control group (*P <* 0.05). However, there was no significant difference in the sputum culture positivity rate between the two groups (*P >* 0.05) (Table-III).

**Table-III:** Comparison of sputum smear and culture results between the two groups.

Group	Sputum smear		Sputum culture	
	Positive	Negative	Positive	Negative
Observation group	56	18	3	69
Control group	47	39	6	81
*t/χ²* value	7.666		0.550	
*P*-value	0.006		0.458	

In the observation group, 94 out of 100 patients (94.00%) tested positive for bacterial pathogens, which was the most common pathogen type, predominantly *Streptococcus pneumoniae* and *Haemophilus influenzae*; 46.00% (46/100) tested positive for viruses, primarily influenza virus and human metapneumovirus; the detection rate of atypical pathogens was also high at 30.00% (30/100); the detection rates of *Mycobacterium tuberculosis* and fungi were low, at 12.00% and 2.00%, respectively. In the control group, 98 out of 118 patients tested positive for pathogens, with the highest detection rate being for bacteria at 68.64% (81/118), primarily *Streptococcus pneumoniae* and *Haemophilus influenzae*; followed by viruses, with a detection rate of 27.97% (33/118), primarily influenza virus and cytomegalovirus; the detection rate of atypical pathogens was lower than that in the observation group, at 15.25% (18/118); the detection rates of *Mycobacterium tuberculosis* and fungi were also low, at 5.24% and 3.39%, respectively. There was a significant difference in the distribution of bacterial, viral, atypical, and *Mycobacterium tuberculosis* pathogens between the two groups (*P <* 0.05, respectively) (Table-IV).

**Table-IV:** Comparison of pathogen detection distribution between the two groups (n[%]).

Pathogen	Observation group (n = 100)	Control group (n = 118)	χ²-value	P-value
Bacterial	94(94.00)	81(68.64)	21.978	0.000
Streptococcus pneumoniae	74(74.00)	64(54.24)		
Staphylococcus aureus	6(6.00)	2(1.69)		
Haemophilus influenzae	12(12.00)	10(8.47)		
Pseudomonas aeruginosa	2(2.00)	3(2.54)		
Klebsiella pneumoniae	0(0.00)	2(1.69)		
Viral	46(46.00)	33(27.97)	7.619	0.006
Influenza virus	38(38.00)	27(22.88)		
Parainfluenza virus	1(1.00)	2(1.69)		
Respiratory syncytial virus	2(2.00)	0(0.00)		
Bocavirus	2(2.00)	0(0.00)		
Human metapneumovirus	3(3.00)	1(0.88)		
Cytomegalovirus	0(0.00)	3(2.54)		
Fungal	2(2.00)	4(3.39)	0.391	0.532
Atypical	30(30.00)	18(15.25)	6.855	0.009
Mycoplasma	18(16.00)	12(10.17)		
Chlamydia	2(2.00)	1(0.88)		
Legionella	10(10.00)	5(4.24)		
Mycobacterium tuberculosis	12(12.00)	5(4.24)	4.536	0.033

After treatment, the observation group had a shorter time to fever resolution, cough resolution, and disappearance of pulmonary wheezing compared to the control group (*P <* 0.05, respectively) (Table-V). It also showed significantly higher SaO_2_ and PaO_2_ levels and a significantly lower PaCO_2_ level compared to the control group (*P <* 0.05, respectively) (Table-VI). Besides exhibiting significantly lower PCT, WBC, CRP, and ESR levels compared to the control group (*P <* 0.05, respectively) (Table-VII).

**Table-V:** Comparison of clinical improvement time between the two groups after treatment (*X̄*±*S*, d).

Group	n	Fever Resolution Time	Cough Resolution Time	Pulmonary Wheezing Resolution Time
Observation group	100	8.02±3.28	19.99±3.31	17.02±3.32
Control group	118	10.72±5.32	22.33±4.73	19.43±4.87
*t*-value		4.418	4.155	4.194
*P*-value		0.000	0.000	0.000

**Table-VI:** Comparison of blood gas parameters between the two groups after treatment (*X̄*±*S*).

Group	n	SaO_2_ (%)	PaO_2_ (mmHg)	PaCO_2_ (mmHg)
Observation group	100	92.92±22.61	96.96±1.49	34.31±4.02
Control group	118	77.74±25.42	94.84±4.58	36.52±6.36
*t-*value		4.617	3.013	4.424
*P*-value		0.000	0.003	0.000

**Table-7:** Comparison of serum inflammatory marker levels between the two groups (*X̄*±*S*).

Group	n	WBC (×10μ/L)	PCT (μg/L)	CRP (g/L)	ESR (mm/h)
Observation group	100	8.34±4.26	0.18±0.53	51.81±56.55	36.06±28.72
Control group	118	10.54±5.53	3.09±12.30	104.66±87.48	59.18±39.64
*t-*value		3.104	2.364	5.191	4.851
*P-*value		0.002	0.019	0.000	0.000

## DISCUSSION

In this study, bacterial, viral, and atypical pathogens were found to be the primary causes of ACALRTIs, which aligns with recent clinical research findings. The proportion of viral CALRTI has been increasing in recent years, particularly with the outbreak of various viral pneumonias over the past decade, leading to a heavy economic burden on society.^7^,^8^ The SARS-CoV-2 pandemic, which began around three years ago, continues to spread globally without signs of slowing down.^9^ Therefore, more attention should be paid to viral CALRTI, and comprehensive viral detection methods should be established to detect and control emerging and sudden infectious diseases. ACALRTIs are common clinical conditions with a wide range of pathogens, which vary across regions, populations, and seasons. Among these pathogens, viruses and bacteria are the most common, while atypical pathogens such as mycoplasma, chlamydia, and fungi are also significant contributors to disease pathogenesis. Identifying the causative pathogen during an ACALRTI episode is fundamental for guiding the appropriate clinical use of antimicrobial treatments.^10^-^12^ However, with the widespread use of antibiotics and advancements in diagnostic technology, the pathogen spectrum of ACALRTIs has evolved. Previously, bacterial pathogens, particularly *Streptococcus pneumoniae* and *Haemophilus influenzae*, were the dominant causes of infection.^13^ In recent years, however, respiratory viruses and atypical pathogens such as mycoplasma and chlamydia have become increasingly prominent. Understanding the pathogen distribution in ACALRTIs^14^-^16^ and establishing efficient treatment strategies to control infection in a short period are critical for improving patient outcomes and enhancing clinical cure rates.

Severe cases of ACALRTIs often present with diminished cough reflex and inability to clear sputum effectively. Some patients experience airway obstruction due to the thickened sputum, exacerbating inflammation and potentially leading to respiratory failure. These patients often require ventilatory support. A crucial aspect of their treatment involves ensuring unobstructed sputum drainage.^17^,^18^ Sputum retention can be resolved through regular suctioning, lavage, and FOB. With the advancement of medical equipment, BAL-FOB has gradually been applied in clinical practice. BAL-FOB effectively removes thick, purulent secretions from the bronchi and alveoli, prevents the absorption of toxins and inflammatory metabolites, ensures airway patency, relieves airway obstruction and hypoxia, increases pulmonary ventilation, and improves the lung’s gas exchange function. The advantages of BAL-FOB^19^,^20^ are as follows:

- The procedure is easy to operate, with minimal adverse reactions.
- The BAL fluid can stimulate the trachea, enhance the cough reflex, and facilitate the clearance of airway secretions, which promotes the reopening of collapsed alveoli and alleviates dyspnea.
- It allows for biopsy and sampling of lung and bronchial lesions, providing valuable insights into lung conditions and enabling the development of effective treatment plans.
- It has broad clinical applications. In this study, the observation group had a higher sputum smear positivity rate compared to the control group, suggesting that the use of FOB to obtain sputum samples in advance increases the likelihood of positive smear results. After BAL-FOB, the observation group exhibited an ORR significantly higher than that of the control group (*P <* 0.05). Additionally, the clinical symptom improvement time in the observation group (fever resolution time, cough resolution time, and disappearance of lung wheezing) was all shorter than in the control group (*P <* 0.05). Furthermore, the observation group showed significantly higher levels of SaO_2_ and PaO _2_than those of the control group. In contrast, the observation group had significantly lower levels of PaCO_2_ and inflammatory markers (PCT, WBC, CRP, and ESR) compared to the control group (*P <* 0.05, respectively).

The reasons for these findings can be explained as follows: BAL-FOB allows for direct visualization and thus maximizes the removal of pulmonary secretions. Additionally, it enables the local delivery of sensitive antibiotics to the infected areas, achieving higher drug concentrations in the target site. This localized high concentration allows the antibiotic to exert its maximum therapeutic effect, providing a strong anti-inflammatory action. Furthermore, this procedure can be repeated multiple times, enhancing its therapeutic effect. It is also minimally invasive, highly safe, targeted, and can be performed even when patients are unconscious.

### Limitations:

Despite the research findings, this study has several limitations. We only perform BAL in patients with complication-free CALRTIs because patients with complications tend to have poorer physical conditions and may not tolerate BAL-FOB well. This might explain the higher pathogen detection rate in the observation group compared to the control group.

## CONCLUSIONS

Bacteria, viruses, and atypical pathogens continue to be the main causative agents of ACALRTIs. In elderly patients with severe ACALRTIs, conventional treatment plus BAL-FOB can significantly improve blood gas parameters, reduce inflammatory markers, and reduce clinical symptoms. This approach can enhance clinical outcomes, and when conditions permit, it should be applied early and multiple times to achieve the best results.

### Authors’ Contributions:

**KG:** Conceived, Literature search, designed the study and review, and is responsible and accountable for the accuracy or integrity of the work.

**SY:** Collected the data, have performed the analysis, critical review, preparation of the manuscript. All authors have read and approved the final manuscript.
